# Evaluation of the Chemical Profile and Antioxidant Capacity of Green, Brown, and Dark Propolis

**DOI:** 10.3390/plants12183204

**Published:** 2023-09-08

**Authors:** Ana Luiza Santos Vieira, Vinícius Tadeu da Veiga Correia, Ana Luiza Coeli Cruz Ramos, Nayana Hayss Araújo da Silva, Leonardo Assis Campos Jaymes, Julio Onésio Ferreira Melo, Ana Cardoso Clemente Filha Ferreira de Paula, Maria Aparecida Vieira Teixeira Garcia, Raquel Linhares Bello de Araújo

**Affiliations:** 1 Department of Food, Faculty of Pharmacy, Campus Belo Horizonte, Federal University of Minas Gerais, Belo Horizonte 31270-901, MG, Brazil; anavieiranutricionista@gmail.com (A.L.S.V.); viniciustvcorreia@gmail.com (V.T.d.V.C.); analuizacoeli@gmail.com (A.L.C.C.R.); nayanahayss@gmail.com (N.H.A.d.S.); leojaymes@outlook.com (L.A.C.J.); mavtgarcia@gmail.com (M.A.V.T.G.); raquel@bromatologiaufmg.com.br (R.L.B.d.A.); 2 Department of Exact and Biological Sciences, Campus Sete Lagoas, Federal University of São João del-Rei, Sete Lagoas 36307-352, MG, Brazil; 3 Department of Agrarian Sciences, Campus Bambuí, Federal Institute of Education, Science and Technology of Minas Gerais, Bambuí 38900-000, MG, Brazil; ana.paula@ifmg.edu.br

**Keywords:** propolis extract, bioactive compounds, PS-MS, antioxidant potential

## Abstract

The chemical composition of propolis varies between different types, due to the specific vegetation found near the hives and the climatic and soil conditions worldwide. Green propolis is exclusive to Brazil, produced by bees, with the resin of the plant *Baccharis dracunculifolia*. Brown propolis is a specific variety produced mainly in Northeast Brazil from the plant *Hyptis divaricata*, also known as “maria miraculosa”. Dark propolis is a variety of propolis produced by bees from the resin of the plant known as Jurema Preta (*Mimosa hostilis benth*). In this study, the aqueous extracts of green, brown, and dark propolis were analyzed for their antioxidant capacity using ABTS, FRAP, and DPPH, and their chemical profiles were determined using paper spray mass spectrometry. Among the three extracts, green propolis had the highest content of total phenolic compounds (2741.71 ± 49.53 mg GAE. 100 g^−1^), followed by brown propolis (1191.55 ± 36.79 mg GAE. 100 g^−1^), and dark propolis had the lowest content (901.79 ± 27.80 mg GAE. 100 g^−1^). The three types of propolis showed high antioxidant capacity, with green showing the highest antioxidant capacity for the three methods used. Using paper spray mass spectrometry, it was possible to suggest the presence of 116 substances, including flavonoids (56), phenylpropanoids (30), terpenes (25), carboxylic acids (1), benzoic acid derivatives (1), fatty acids (1), amino acids (1) and alkaloids (1). The compounds in the green, brown, and dark propolis extracts reinforce the bioactive potential for application in these tree extracts’ food and pharmaceutical products.

## 1. Introduction

Propolis, also called “bee glue”, is a natural product collected from different species of bees, among them *Apis mellifera*. It is produced through the exudates collected from shoots and flowers of different plant species that grow near the hive [1,2,3]. These exudates are transported to the hive, chewed by the bees, and mixed with the bee’s pollen and saliva, containing several enzymes. Finally, after adding beeswax, crude propolis is formed, composed of approximately 50% resins, 30% wax, 10% essential oils, 5% pollen, and 5% other substances and materials, including organic compounds [4,5,6]. Human beings have used propolis since the earliest civilizations. Priests in ancient Egypt continually used propolis as a medicinal substance and an integral part of embalming cream essences. Later, Persians, Romans, and Incas used propolis to treat infections, as oral disinfectants, and as an antiseptic and healing agent in treating wounds [7,8,9,10].

The physicochemical properties (density, color, odor), chemical profile, and biological activity of a given propolis sample are determined by some factors, including plant origin (precursor), climate, climatic conditions of the harvest year, and sometimes the time of harvest [1]. It is also classified according to the flora where the bees collect the resins, representing the raw material for propolis production [11].

The Brazilian green propolis comes from the plant *Baccharis dracunculifolia*, also popularly known as “rosemary of the field”. It is native to Brazil’s Southeast and South regions, and has been the subject of several studies for medicinal, phytochemical, and pharmacological purposes [12,13,14].

This variety of propolis has more than 200 identified chemical compounds. Among them, the polyphenolic compound artepellin C (3,5-diprenyl-4-hydroxycinnamic acid) is considered as the main bioactive compound, and the phenolic compounds bacarin and drupanin are described as chemical markers for this type of propolis, originating in *B. dracunculifolia* [14,15,16].

As far as brown propolis is concerned, it is mainly produced in Northeast Brazil from *H. divaricata* [17]. Dembogurski et al. [18] described in their research the characterization of brown propolis, composed of flavonoids, fatty acids and phenylpropanoid acid, and its prenylated derivatives, such as artepillin C.

This type of propolis is characterized by a flavonoid-rich composition without B ring substituents (e.g., pinocembrin, pinobanksin, galangin, chrysin) and its esters, along with phenylpropanoids and their esters (e.g., phenylethyl ester of caffeic acid, CAPE) [16].

Dark propolis is produced by bees from the resin of the plant called Jurema Preta (*Mimosa Hostilis benth*), a northeastern tree from Brazil [19]. In their research, Oliveira et al. [20] reveal that 3,4-dihydroxybenzoic acid, rutin, and trans-cinnamic acid are the main chemical compounds responsible for antioxidant and antibacterial activity. Evidence from in vitro and in vivo studies corroborates its pharmacological effects: antioxidant, anti-inflammatory, antimicrobial, antidiabetic, antitumor, neuroprotective, gastroprotective, and immunomodulatory [21].

Recent studies have shown the promising antioxidant activity of propolis through the mechanisms of elimination, neutralization, and the removal of reactive species after the induction of oxidative stress, and by preventing lipid peroxidation [22,23].

There are different methods of evaluating the antioxidant capacity of a product. Several in vitro methods have been applied to evaluate the antioxidant capacity of plant products. Combining at least two in vitro methods is recommended to produce more reliable information on the total antioxidant capacity of a food. The iron reduction method FRAP (ferric reducing antioxidant power), ABTS+ free radical capture (2,2′-azinobis (3-Ethylbenzothiazoline-6-sulfonic acid), and DPPH free radical capture DPPH (2,2-difenil-1-picril-hidrazil) are, according to the literature, the most widely used to determine antioxidant capacity in vitro [11,24].

Several techniques can be used for a more detailed characterization of the antioxidant compounds present in plant species, such as gas chromatography and liquid chromatography, which quantify the compounds, combined or not, with mass spectrometry (MS), which identifies the profile of the compounds in the samples. The ambient ionization paper spray (PS) stands out among the identification techniques [25,26].

Paper spray mass spectrometry (PS-MS) is an efficient and low-cost technique for analyzing substances in complex matrices. It allows for the rapid obtaining of fingerprints of different samples without generating chemical residues. The technique uses high-voltage spray ambient ionization to record the analytes of a solvent extract on paper [26].

PS-MS is similar to the electrospray ionization (ESI) technique, and has been applied in fraud detection, pesticide analysis, and phytochemical characterization of various foods. This technique provides a fast and versatile approach to complex sample analysis, with a wide mass range and minimal sample preparation [27].

The advantages of paper spray mass spectrometry range from greater replicability to shorter data acquisition time for greater signal stability. In this method, a solvent extracts the compounds present in the raw material. Hence, the drag of the extracted analytes is recorded on paper and a spray ionization is used, due to the high voltage applied [28].

Propolis is, therefore, an important source of bioactive compounds, with potential beneficial effects on human health. However, there are no reports on the presence and comparative data of the types of phenolic compounds and antioxidant activity among the different varieties of Brazilian propolis. Thus, it is essential to investigate the chemical composition of different species and their comparative evaluation [27]. In this context, when evaluating the antioxidant activity and the chemical profile of green, brown and dark propolis from the Serra da Canastra region, Bambuí, Minas Gerais, the study seeks to contribute to the scientific knowledge of these natural products and their possible application in medicine and health and wellness products.

## 2. Results and Discussion

### 2.1. Total Phenolic Compounds and Antioxidant Capacity

The content of total phenolic compounds and the antioxidant capacity of the samples of green, brown and dark propolis, according to the ABTS, FRAP, and DPPH methods, are presented in Table 1.

Different tests were applied to evaluate the antioxidant capacity of the extracts of green, brown, and dark propolis, which allows a comprehensive evaluation of the antioxidant potential of the samples.

The chemical composition of propolis is very diverse and complex. This chemical composition depends on factors such as the flora around the hive accessible to bees, collection time, and diversity of trees and plant species collected by bees [15,29], which justifies the different contents of total phenolic compounds among the three propolis types studied.

The contents of total phenolic compounds of the propolis extracts were 2741.71 ± 49.53 mg GAE/100 g for the green propolis extract, 1191.55 ± 36.79 mg GAE/100 g for the brown propolis extract and 901.79 ± 27.80 mg GAE/100 g for the dark propolis extract.

These results showed that the green propolis extract presented the highest content of phenolic compounds among the three extracts. This type of propolis, from the plant source *Baccharis dracunculifolia*, is Brazil’s primary source of green propolis, and is characterized by its high content of total phenolic compounds [29,30].

Berretta et al. [23], when studying the content of total phenolic compounds in Brazilian green propolis in three different formulations osf propolis extract (polar fraction of propolis, dry extract of soluble propolis and microencapsulated propolis extract), found values ranging from 2258 to 6162 mg GAE/100 g. The present study found a value of 2741.71 ± 49.53 mg GAE/100 g for the green propolis extract.

The difference between the total phenolic compounds’ contents can be explained due to the different technologies used to produce the extracts, which affect their physical appearance, chemical profile, and biological activity.

Barbosa et al. [31] determined the content of phenolic compounds in propolis produced in Roraima by the honey bee *Apis*. They found 639 and 4089 mg GAE/100 g for forest and savanna samples, respectively, in which the researchers used the ethanolic extract of propolis. This may explain the difference in the results when compared to those found in this study, which used the aqueous extract of propolis.

The propolis collection region can also influence its composition. Because of the specific vegetation found in the areas close to the beehives and the climatic and soil conditions, the propolis from Roraima (the Northeast region of Brazil) does not come from the same region as the propolis in this study.

Regarding the content of total phenolic compounds in brown propolis, an intermediate value was found (2741.71 ± 49.53 mg GAE/100 g), which is lower than the amount found in the extract of green propolis (1191.55 ± 36 79 mg GAE/100 g) and higher than the amount found in the dark propolis extract (901.79 ± 27.80 mg GAE/100 g).

In a study that evaluated the content of total phenolic compounds of brown propolis from the state of Santa Catarina, the value found was 873.3 mg GAE/100 g, a value lower than that found in the present study for brown propolis, which was 1191.55 mg GAE/100 g [32].

This variation can be explained by the different regions of origin of brown propolis between the two studies and by the variation in the extraction processes used by the researchers. In the study analyzed, the researchers used a method in which they obtained a hydroalcoholic extract as a final product.

Propolis has a high antioxidant capacity, determined by its phenolic compounds [3].

Considering different botanical origins, seasonality, and different extraction methods between the present study and others that evaluated the antioxidant capacity of different types of propolis, it was verified that the results of the antioxidant capacity described in the literature are varied, as presented below.

Regarding the results in Table 1, the green propolis sample presented the highest antioxidant capacity among the others in all the methods applied.

The antioxidant capacity of propolis may vary depending on its chemical composition, influenced by the plants in the region of origin. Therefore, each variety of propolis has a different composition, and there may be propolis with a greater antioxidant capacity [33].

Studies indicate that some varieties of propolis have demonstrated a more pronounced antioxidant capacity. For example, Brazilian green propolis, especially that from the *Baccharis dracunculifolia* plant, is known to have a high content of artepillin C, a phenolic compound with an intense antioxidant capacity [29,33,34].

Andrade et al. [33] also reported a higher antioxidant capacity of green propolis (ABTS: 2214.96 μM Trolox/g; FRAP: 604.20 μM ferrous sulfate/g) when compared to brown propolis (ABTS: 1868.45 μM Trolox/g; FRAP: 471.51 μM ferrous sulfate/g).

Regarding the DPPH method, a lower EC50 valuse indicates a more significant antioxidant capacity, since a smaller extract mass is needed to inhibit 50% of the DPPH radical]. The present study found a greater antioxidant capacity of green propolis (491.68 ± 44.55 EC50 μg g^−1^ for DPPH) when compared to that reported by Skaba et al. [35] (1230.07 EC50 μg g^−1^ for DPPH).

Using the FRAP method, the value found for green propolis was 422.83 μM of ferrous sulfate/g. Mello and Hubinger [36], when studying the antioxidant capacity of Brazilian green propolis in the State of São Paulo, reported values ranging from 180.95 to 1038.09 for the aqueous extract in different pH ranges.

This difference can be explained by the region of origin of the propolis and by the influence of the extraction method with pH modification.

In a study that evaluated the antioxidant capacity of Brazilian green propolis using the FRAP method, Casagrande et al. [37] found a value of 458.75 μM of ferrous sulfate/g, very close to that reported in the present study.

Andrade et al. [38], when applying the FRAP method to evaluate the antioxidant capacity of green and brown propolis, found 293.49 μM of ferrous sulfate/g and 213.76 μM of ferrous sulfate/g, respectively. In the present study, the results were 422.83 μM of ferrous sulfate/g for green propolis and 179.54 μM of ferrous sulfate/g for brown propolis.

Another study, which evaluated the antioxidant capacity of 47 samples of propolis collected in different locations, perhaps in the Black Sea region of Turkey, including dark propolis, found an average value of 112.98 μM of ferrous sulfate/g. This result was lower than that found in this study for dark propolis, using the FRAP method (161.29 ± 2.59 μM ferrous sulfate/g) [39].

Salgueiro and Castro [40], when studying extracts of green propolis in natura and commercially, reported the following values: 84.86 ± 0.01 to 246.83 ± 0.05 μM of Trolox/g for ABTS. The present study found a value of 293.90 μM of Trolox/g, using this same method.

Several factors may explain the difference between the results found in the present study and those reported in the literature, such as botanical source, region of origin of propolis, seasonality, and extraction method.

The type of propolis is named based on the plant source, and exerts influence on its composition. Thus, depending on the type of propolis, its chemical composition differs, which can result in differences in antioxidant capacity [39].

The concentration and composition of propolis extract depend on the choice and effectiveness of the extraction method used. Processing parameters such as sample–solvent ratio, temperature, and extraction time significantly affect the diversity and concentration of compounds in the final extract [41,42].

### 2.2. Chemical Profiling using Paper Spray Mass Spectrometry (PS/MS)

The green, brown, and dark propolis extracts were analyzed using paper spray ionization with mass spectrometry (PS-MS) in the positive and negative ionization modes. The analysis of the spectra of the aqueous extracts of green, brown, and dark propolis indicated that the negative ionization mode provided a higher sensitivity level than the positive one, allowing the identification of several compounds. The positive ionization mode allowed the possible identification of 18 compounds (Table 2) and 98 compounds in the negative ionization mode (Table 3), with a total of 116 compounds possibly identified.

The complete PS(+)MS and OS(-)MS scan of dark, green and brown propolis aqueous extract can be found in the Supplemental Material.

The ions and their fragments obtained in this analysis were identified based on the data described in the literature. The possible compounds identified belong to the following chemical classes: flavonoids (56), phenylpropanoids (30), terpenes (25), carboxylic acids (1), benzoic acid derivatives (1), fatty acids (1), amino acids (1) and alkaloids (1).

**Table 2:** Chemical profile of green, brown, and dark propolis extracts using Paper Spray Mass Spectrometry (PS/MS) in positive mode.

*m*/*z*	Attempted Identification	Chemical Class	MS/MS	Type of Propolis			References
				Green	Brown	Dark	
285	Biochanin A	Flavonoid	213, 225, 229, 242, 253, 257, 270, 285	X		X	[43,44]
	Galangin-5-methyl-ether	Flavonoid	239, 270	X		X	[5]
	Methoxy-chrysin	Flavonoid	242, 257, 270	X		X	[5]
	Galangin-5-methyl-ether	Flavonoid	270	X		X	[5]
	Homopterocarpin	Flavonoid	149, 163, 257, 270, 285	X		X	[44]
	7,3′-Dihydroxy-5′-Methoxy-isoflavon	Flavonoid	225, 229, 253, 257	X		X	[45]
	Caffeic acid phenylethyl ester (CAPE)	Phenylpropanoid	163	X		X	[5]
301	Artepillin C	Phenylpropanoid	203, 245, 269	X	X		[46]
317	Quercetin-3-methyl-ether	Flavonoid	285, 302	X	X		[5]
	Isorhamnetin	Flavonoid	257, 285, 302	X	X		[5]
	Quercetin-7-methyl-ether	Flavonoid	167, 243, 261, 271, 302	X	X		[5]
	(3S)-violanone	Flavonoid	271, 289, 299	X	X		[44]
	7-hydroxy dehydroabietic acid	Terpene	215, 243, 271, 275, 299	X	X		[47]
331	Quercetin-dimethyl-ether	Flavonoid	316	X			[5]
340	Lobelanidin	Alkaloid	202, 322			X	[47]
371	Pinobanksin-3-*O*-hexanoate	Flavonoid	227, 255, 273	X			[5]
771	Scopoloside II	Terpene	762	X	X		[48]

The MS/MS fragments represented on the same line as the *m/z* ions refer to the same ion.

Of the possible compounds identified in the extracts of green, brown, and dark propolis with the positive ionization mode, most belong to the flavonoid class (12), but there are also compounds belonging to the terpene (2), phenylpropanoid (2) and alkaloids (1).

Among these classes, the flavonoids were more significant in the three analyzed extracts: 13 in green propolis, 7 in the dark, and 4 in brown.

Flavonoids are important for their antioxidant properties, which can bind to free radicals and protect cells from lipid peroxidation. They also exhibit anti-inflammatory properties [49].

In a comparative study of green propolis from different Brazilian states, Righi et al. [49] reported phenolic compounds and flavonoids as the leading chemical classes, which corroborates the chemical profile of this study.

Flavonoids and other phenolic compounds protect cells from damage caused by oxidation by acting as potent inhibitors of oxidative stress, which is involved in the pathogenesis of neurodegenerative diseases [50].

In addition, the phenolic compounds’ intracellular free radical scavenging capabilities can protect cell membranes against lipid peroxidation [51].

Phenolic compounds exert antioxidant capacity by donating hydrogen atoms from an aromatic hydroxyl group, leading to the sequestration of free radicals [52].

Funari et al. [53] determined the chemical profile of Brazilian green propolis. The total flavonoid and phenolic compounds were determined using spectrophotometry, and the chemical composition, using HPLC, was characterized mainly by flavonoids and aromatic acids. The compounds identified in the ethanolic extract and methanolic extract of propolis were artepillin C, coumaric p-acid, ferulic acid, trans-cinnamic acid, chlorogenic acid, caffeic acid, kaempferol, kaempferide and isosakuranetin. Most of these compounds were also identified in this study.

Berretta et al. [23], when studying Brazilian green propolis, reported the presence of galangin, artepillin C, and baccarin, compounds that were also tentatively identified in the green propolis extract analyzed in this study.

The phenolic acid at *m*/*z* 301, artepillin C, is a prenylated derivative of *p*-coumaric acid, isolated from the *Baccharis* species, and is one of the main phenolic compounds found in Brazilian green propolis, which adds high value to this bee product. It is a major and unique component of this type of propolis. It is responsible for various beneficial health properties, such as antioxidant, anti-inflammatory, antidiabetic, neuroprotective, gastroprotective, immunomodulatory, and anticancer effects [21]. Bees collect exudates from *Baccharis dracunculifolia* to produce green propolis, which contains a high concentration of this compound [54].

Although green propolis contains the highest level of artepillin C as the main compound of *Baccharis dracunculifolia*, various amounts have also been reported in Brazilian brown propolis [55]. These reports in the literature corroborate the result found in the present study, which identified artepillin C in green and brown propolis extracts.

The compound artepillin C has gained immense attention globally, and therefore green propolis has achieved a high commercial value on the global market. Propolis containing this compound is considered high quality [54,55].

In the present study, two compounds belonging to the terpene class were identified in the positive ionization mode in the brown propolis extract: sugiol (*m*/*z* 301) and 7-hydroxy dehydroabietic acid (*m*/*z* 317), and in a study that analyzed the chemical profile of Brazilian brown propolis, the most abundant constituents belonged to the terpene class [56].

Alamo-like propolis, prevalent in the northern hemisphere, contains various phenolic components, including aromatic aldehydes, flavonoids and phenolic acids, and their esters, such as the phenyl esters of caffeic acid [57].

In a study that evaluated the chemical profile of poplar-type propolis from the Black Sea region (Turkey), caffeic acid, quercetin and CAPE were identified as dominant compounds. The authors also emphasized that CAPE is the characteristic marker of black poplar propolis, in addition to flavonoid aglycones [58]. This compound was also identified in dark propolis in the present study.

In a study that determined the chemical profile of essential oils of Polish propolis and black poplar, the authors found as main chemical classes free phenolic acids, flavonoids, and monoesters of phenolic acids and flavonoids [59], a profile similar to that found in the present study for dark propolis.

**Table 3:** Chemical profile of green, brown, and dark propolis extracts using Paper Spray Mass Spectrometry (PS/MS) in negative mode.

*m*/*z*	Attempted Identification	Chemical Class	MS/MS	Type of Propolis			References
				Green	Brown	Dark	
163	*p*-Coumaric acid	Phenylpropanoid	119			X	[5]
	*m*-Coumaric acid	Phenylpropanoid	119			X	[57]
	2-Hydroxycinnamic acid	Phenylpropanoid	119			X	[57]
173	Shikimic acid	Carboxylic acid	93	X			[57]
179	Caffeic acid	Phenylpropanoid	179,135	X		X	[9,60]
191	Quinic acid	Phenylpropanoid	59, 85, 93, 127, 176, 191	X	X	X	[9,57]
231	Drupanin	Phenylpropanoid	132, 187, 231	X		X	[61]
	*p*-Coumaric prenyl ester	Phenylpropanoid	163	X		X	[5]
247	Caffeic acid prenyl ester	Phenylpropanoid	179	X		X	[5]
253	Chrysin	Flavonoid	165, 181, 209, 253			X	[5]
	Chrysin isomer (I)	Flavonoid	209, 253			X	[60]
	*p*-Coumaric benzyl ester	Phenylpropanoid	162, 145			X	[5]
255	liquiritigenin—Isoliquiritigenin	Flavonoid	193, 200, 227, 255	X	X	X	[62,63]
	Pinocembrin	Flavonoid	171, 211, 227, 255	X	X	X	[9,64]
267	Formononetin	Flavonoid	267, 268			X	[63]
	Chrysin-5-methyl-ether	Phenylpropanoid	195, 224			X	[5]
269	Medicarpin	Flavonoid	197, 225, 241, 251, 254, 269	X	X	X	[62,63]
	Apigenin	Flavonoid	181, 183, 197, 201, 225, 227	X	X	X	[9]
	Caffeic acid benzyl ester	Phenylpropanoid	134, 225	X	X	X	[9]
271	Pinobanksin	Flavonoid	107, 151, 169, 185, 209, 215, 225	X		X	[5,64,65]
	Naringenin	Flavonoid	93, 107, 121, 151, 177, 225, 253	X		X	[9,60]
	Neovestitol	Flavonoid	271	X		X	[63]
	Vestitol	Flavonoid	271	X		X	[63]
283	Biochanin A	Flavonoid	227, 255, 268, 269, 284	X		X	[62,63]
	Galangin-5-methyl-ether	Flavonoid	211, 239, 240, 268	X		X	[5,64]
	Methoxy-chrysin	Flavonoid	211, 239, 268	X		X	[5]
	Caffeic acid phenylethyl ester (CAPE)	Phenylpropanoid	135, 179	X		X	[65]
285	Luteolin	Flavonoid	132, 151, 285	X	X	X	[65]
	Vestitone	Flavonoid	151, 270, 285	X	X	X	[63]
	Sakuranetin	Flavonoid	150, 165	X	X	X	[9]
289	(+)-Catechin—(±)-Epi-catechin	Flavonoid	245			X	[57]
295	Caffeic acid cinnamyl ester	Phenylpropanoid	159, 251	X		X	[9]
299	Kaempferide	Flavonoid	200, 255, 256, 284	X	X	X	[9,66]
	Luteolin-methyl-ether	Flavonoid	255, 256, 284, 285, 299	X	X	X	[5,64,65]
	Diosmetin	Flavonoid	284, 285, 299	X	X	X	[65]
	Dehydroabietic acid	Terpene	299, 300	X	X	X	[65]
301	Dihydrokaempferide	Flavonoid	107, 125, 151, 152, 180, 255	X	X	X	[66]
	Quercetin	Flavonoid	107, 151, 179, 229, 245, 257	X	X	X	[5,9]
	Hesperetin	Flavonoid	151, 257, 268, 286	X	X	X	[65]
	Trans-communic acid	Terpene	301, 302	X	X	X	[65]
	Sternbin	Flavonoid	165, 223, 239, 255, 268, 283	X	X	X	[9]
	Diterpene acid	Terpene	257, 268, 273, 283, 286	X	X	X	[9]
	Ellagic acid	Benzoic acid derivative	229, 284	X	X	X	[57]
	Eicosapentaenoic acid	Fatty acid	301	X	X	X	[67]
303	Taxifolin	Flavonoid	125			X	[57]
305	Epigallocatechin	Flavonoid	125, 219		X		[57]
311	Caffeic acid 4-*O*-arabinoside	Phenylpropanoid	183	X		X	[49]
	Coumaric acid derivative	Phenylpropanoid	119, 163, 267	X		X	[9]
313	Pinobanksin-3-*O*-acetate	Flavonoid	185, 225, 254	X	X	X	[9,60]
	Ermanin	Flavonoid	255, 283, 298	X	X	X	[64]
315	Isorhamnetin	Flavonoid	163, 241, 243, 253, 255, 271, 300	X	X	X	[9,49,57]
	Quercetin-3-methyl-ether	Flavonoid	243, 245, 271, 300	X	X	X	[5,64]
	Quercetin-7-methyl-ether	Flavonoid	243, 271, 300	X	X	X	[64]
	Rhamnetin	Flavonoid	271, 287, 300	X	X	X	[9]
	5,4′-dihydroxy-7,3′-dimethoxyflavanone	Flavonoid	271, 300	X	X	X	[9]
	Caffeic acid derivative	Phenylpropanoid	271	X	X	X	[9]
	Diterpene acid	Terpene	253, 297	X	X	X	[9]
	Hydroxydehydroabietic acid isomer (I)	Terpene	253, 315, 316	X	X	X	[65]
	Hydroxydehydroabietic acid isomer (II)	Terpene	297, 315, 316	X	X	X	[60]
316	3-*O*-methylquercetin	Flavonoid	187, 301	X		X	[57]
317	Myricetin	Flavonoid	179	X		X	[57]
	Hydroxyisopimaric acid isomer (I)	Terpene	225, 317, 318	X		X	[65]
	Hydroxyisopimaric acid isomer (II)	Terpene	299, 300, 317, 318	X		X	[65]
	Hydroxyisopimaric acid isomer 4	Terpene	317, 318	X		X	[65]
319	Cupressic acid isomer 1	Terpene	319, 320			X	[65]
	Cupressic acid isomer 2	Terpene	287, 319, 320			X	[65]
	Diterpene acid	Terpene	275, 301			X	[9]
327	Pinobanksin-5-methyl-ether-3-*O*-acetate	Flavonoid	165, 195	X		X	[5]
	Pinobanksin-3-*O*-propionate	Flavonoid	165, 197, 199, 209, 227, 253, 254	X		X	[5,9,65]
	Caffeic acid derivative	Phenylpropanoid	133, 283	X		X	[9]
329	Quercetin-dimethyl-ether	Flavonoid	239, 255, 271, 285, 286, 299, 300	X	X	X	[5,9,64]
	Ferulic acid dihydroxy phenyl ethyl ester	Phenylpropanoid	285, 299, 314	X	X	X	[49]
	Artepillin C derivative	Phenylpropanoid	255, 299	X	X	X	[67]
331	Laricitin	Flavonoid	229, 261, 287, 313	X	X	X	[49]
	3,5,4′-trihidroxy-7,3′-dimetoxiflavanone	Flavonoid	179, 271, 273, 288, 303, 313	X	X	X	[9]
	Dihydroxydehydroabietic acid isomer (I)	Terpene	331, 332	X	X	X	[60]
	Dihydroxydehydroabietic acid isomer (II)	Terpene	313, 314, 331, 332	X	X	X	[60]
333	Agathic acid isomer (I)	Terpene	333, 334			X	[60]
	Agathic acid isomer (II)	Terpene	333, 334			X	[60]
	Agathic acid isomer (III)	Terpene	315, 333, 334			X	[60]
	Agathic acid isomer 1	Terpene	333, 334			X	[65]
	Agathic acid isomer 4	Terpene	333, 334			X	[65]
345	Eupatolitin	Flavonoid	223, 237, 285, 286, 287, 301, 312	X	X	X	[9]
347	Diterpene acid	Terpene	201, 271, 273, 315	X	X	X	[9]
353	Chlorogenic acid	Phenylpropanoid	173, 179, 191	X	X	X	[57]
355	Pinobanksin-3-*O*-pentanoate	Flavonoid	180, 181			X	[65]
357	Matairesinol	Phenylpropanoid	342, 357			X	[60]
361	(-)-Secoisolariciresinol	Phenylpropanoid	346, 361	X		X	[60]
	15-Acetoxy-cupressic acid	Terpene	361, 362,	X		X	[65]
469	Cycloartane triterpene acid	Terpene	351, 383, 391, 407, 408, 423, 425		X		[9]
471	Cycloartane triterpene acid	Terpene	339, 359, 391, 393, 403, 407, 409		X		[9]
515	Dicaffeoylquinic acid isomer 1	Phenylpropanoid	179, 191, 353	X	X	X	[65]
	Dicaffeoylquinic acid isomer 2	Phenylpropanoid	179, 191, 353	X	X	X	[65]
	Dicaffeoylquinic acid isomer 3	Phenylpropanoid	173, 179, 191, 353	X	X	X	[65]
	DiCaffeoylquinic acid	Phenylpropanoid	173, 179, 191, 203, 255, 299, 335	X	X	X	[9]
	DiCaffeoylquinic acid isomer	Phenylpropanoid	173, 179, 191, 203, 299, 353	X	X	X	[9]
677	Tricaffeoylquinic acid	Phenylpropanoid	299, 317, 353, 497, 515	X	X	X	[49]
747	Leucine-aspartate-lysine	Amino acid	419, 567	X			[48]

The MS/MS fragments represented on the same line as the *m/z* ions refer to the same ion.

The possible compounds identified in the extracts of green, brown and dark propolis with the negative ionization mode belong mainly to the classes of flavonoids (42), phenylpropanoids (28), and terpenes (19).

Regarding these classes among the three types of propolis studied, they are divided as follows: green propolis: 36 flavonoids, 22 phenylpropanoids and 13 terpenes; brown propolis: 26 flavonoids, 12 phenylpropanoids, and 11 terpenes; and dark propolis: 42 flavonoids, 28 phenylpropanoids and 21 terpenes.

Guimarães et al. [68] investigated the antioxidant properties of Brazilian green propolis, and mainly identified compounds belonging to the classes of flavonoids and phenolic compounds. Caffeic, *p*-coumaric, trans-cinnamic acids, aromadendrin-4-*O*-methyl ether, isosakuranetin, quercetin, and kaempferol were identified.

Among these, caffeic acid, *p*-coumaric acid, cinnamic acid, quercetin and kaempferol were also possibly identified in the present study for the negative ionization mode regarding the green propolis extract. The chemical profile presented in this study corroborates the similarity between the compounds identified in other studies of green propolis [68,69,70].

Berretta et al. [23], when studying Brazilian green propolis, reported the presence of the compounds caffeic acid and *p*-coumaric acid, decaffeoylquinic acid, drupanin, chrysin, and galangin in the analyzed sample, compounds also provisionally identified in the green propolis extract analyzed in the present study.

Falcão et al. [16], when analyzing the chemical profile of Brazilian green propolis, identified 21 phenolic compounds, six phenolic acids (of which p-coumaric acid; *m*/*z* 163, and dicaffeoylquinic acid; *m*/*z* 515, were the most representative) and four flavonoids (which included kaempferol; *m*/*z* 285, and kaempferide and its isomer of kaempferide; *m*/*z* 299). The chemical profile reported by these researchers was very similar to that found in the present study for the same type of propolis.

Green propolis is widely researched concerning other types of propolis. Due to this, there is a higher number of available data on this type of propolis, compared to those of brown and dark propolis.

Falcão et al. [16], when evaluating the chemical profile of brown propolis, reported a high number of compositions belonging to the classes of flavonoids and phenylpropanoids, which corroborates the result found in the present study, where the main chemical classes of brown propolis extract were flavonoids, terpenes, and phenylpropanoids.

In the present study, 11 compounds belonging to the terpene class were provisionally identified in the negative ionization mode in the brown propolis extract, namely: dehydroabietic acid (*m*/*z* 299); trans-communic acid and diterpenic acid (*m*/*z* 301); isomer of hydroxysidroabietic acid (I); isomer of hydroxysidroabietic acid (II) and diterpene acid (Terpene) (*m*/*z* 315); isomer of hydroxysidroabietic acid (I) and isomer of hydroxysidroabietic acid (II) (*m*/*z* 331); diterpenic acid (*m*/*z* 347); cycloartane acid triterpenes (*m*/*z* 469) and cycloartane acid triterpenes (*m*/*z* 471).

In a study that analyzed the chemical profile of Brazilian brown propolis, the most prevalent class was terpenes [56].

Plants produce terpenes for interactions with other organisms. Terpenes protect plants against pathogens such as mold, fungi, and bacteria, and can attract pollinating insects or repel herbivores [71]. When ingested through their botanical sources by humans, they provide various medicinal properties, including antimicrobial, antioxidant, anticancer, antiarrhythmic, anesthetic, anti-inflammatory, antihistamine, antispasmodic, antitumor, antidiabetic and antimicrobial [72].

Some terpenes, such as limonene, linalool, and beta-caryophyllene, have anti-inflammatory properties. They can help reduce the production of inflammatory mediators and modulate the immune system response, decreasing inflammation in various conditions [73].

Many terpenes exhibit antioxidant properties, which means they can neutralize free radicals and reduce oxidative stress in the body. These properties can help protect cells and tissues from damage caused by free radicals, contributing to overall health and disease prevention [74].

In a study that evaluated brown propolis, 39 identified phenolic compounds were reported. Among them, the benzyl ester of caffeic acid and apigenin (*m*/*z* 269), quercetin-3-methylether and isorhamnetin (*m*/*z* 315), kaempferol (*m*/*z* 299), galangin-5-methylether (*m*/*z* 283, benzyl ester of caffeic acid (*m*/*z* 269), pinocembrin (*m*/*z* 255) and pinobaskin-3-o-acetate (*m*/*z* 313) were possibly identified [16]. The present study also identified these same compounds when analyzing the brown propolis extract.

Dark propolis is a specific variety of propolis, with a distinct chemical profile compared to others. Its chemical profile can vary, depending on factors such as the region of origin, the plants that are the sources of the resin, and environmental conditions. Dark propolis is rich in phenolic compounds, including flavonoids, phenolic acids, and their derivatives.

In the present study, compounds that were found exclusively in dark propolis were possibly identified, such as lobelanidin, *p*-coumaric acid, 2-hydroxycinnamic acid, *m*-coumaric acid, chrysin, *p*-coumaric benzyl ester, chrysin isomer (I), chrysin-5-methyl-ether, formononetin, (+)-catechin—(±)-epi-catechin, taxifolin, cupressic acid isomer 1, cupressic acid isomer 2, diterpene acid, agathic acid isomer (I), agathic acid isomer (II), agathic acid isomer (III), agathic acid isomer 1 (carboxylic acid), agathic acid isomer 4, pinobanksin-3-*O*-pentanoate and matairesinol.

These compounds are divided into flavonoids, terpenes, carboxylic acids, phenolic acids, alkaloids, phenylpropanoids and hydroxynamic acids.

Among these, *p*-coumaric acid (*m*/*z* 163), provisionally identified in the negative ionization mode, has anti-inflammatory properties. In a study that evaluated the effects of *p*-coumaric acid on various inflammatory parameters, it was concluded that treatment with *p*-coumaric acid not only reduced the levels of inflammatory mediators (cytokines and lipids) but also increased the production of the anti-inflammatory cytokine IL-10 [75].

In another study, the botanical origin of Brazilian propolis was investigated using high-performance reverse-phase thin-layer chromatography (RP-HPTLC), high-performance reverse-phase liquid chromatography (RP-HPLC), and gas chromatography–mass spectrometry (GCMS) [17]. Several compounds were identified, including derivatives of hydroxycinnamic acid (coumaric acid and ferulic acid—*m*/*z* 119) and flavonoids (pinobanksin—*m*/*z* 271 and kaempferol—*m*/*z* 313), compounds also identified in the present study.

## 3. Materials and Methods

### 3.1. Material

The samples of green, brown, and dark propolis (resins) used in the research were donated by the company Bee Propolis Brasil from apiaries, whose native flora is rich in rosemary from the field (*Baccharis dracunculifolia*), collected in the Serra da Canastra in the municipality of Bambuí, Minas Gerais, (Latitude: 20°1′17″ South, Longitude: 45°57′39″ West) in 2022. The samples were stored separately in a freezer at −18 °C until the moment of analysis.

### 3.2. Methods

#### 3.2.1. Obtaining the Extracts

The aqueous extract was chosen to be used in the present study, due to the toxicity of methanol and the need for an additional rotary evaporation step for the alcoholic extract. This reduces the number of steps in the extraction process, plus the fact that the aqueous extract is the most suitable for ingestion.

The extraction of the compounds from the propolis samples was performed according to Rufino et al. [24], with modifications, to determine the content of phenolic compounds, evaluate the antioxidant capacity and analyze the chemical profile. To obtain the propolis extracts, 1.0 g of each sample was weighed in a 50 mL Falcon tube, to which 10 mL of distilled water was added. The samples were stirred in a vortex stirrer for 30 s and kept at rest for 1 h at room temperature (25 °C). Subsequently, centrifugation was performed in a centrifuge (Mod Jouan BR4) for 20 min with a rotation of 25,407× *g*. The supernatant was transferred to microtubes (2 mL), and the extracts were stored at freezing temperature (−18 °C) until the analysis. The entire extraction process and the antioxidant capacity analyses were carried out under the cover of light and in triplicate.

#### 3.2.2. Determination of the Content of Total Phenolic Compounds

The Folin–Ciocalteau spectrophotometric method was used [76], with some modifications, to determine the total phenolic compound content of green, brown, and dark propolis extracts. The extracts were diluted in 70% acetone solution (*v*/*v*) in 10 mL falcon tubes covered with aluminum foil, and 5 mL of 10% Folin–Ciocalteau solution was added. This mixture was homogenized in a tube agitator (Nova instruments, NI 1066, Piracicaba, SP, Brazil) for 5 s, and kept at rest for 5 min. After this period, 4 mL of 7.5% sodium carbonate solution (*w*/*v*) was added, and the tubes were incubated at room temperature for 60 min. After this incubation, the sample was read at 760 nm in a UV-visible absorption spectrophotometer (Analytik Jena, Spekol 1300, Jena, Germany), using 70% (*v*/*v*) acetone solution as white. The result was expressed as gallic acid equivalent (mg GAE/100 g of sample).

#### 3.2.3. Determination of Total Antioxidant Capacity

Three methods determined the total antioxidant capacity of green, brown, and dark propolis extracts: (1) reaction with 2,2′-diphenyl-1-picrilhidrazil (DPPH); (2) capturing the free radical ABTS*+; and (3) iron reduction reaction (FRAP, Ferric Reducing Antioxidant Power).

##### Antioxidant Capacity by Reaction with DPPH Free Radical

The DPPH analysis was performed according to the official method 2012.04 [77], with some adaptations. Three different dilutions in triplicate were prepared in 10 mL falcon tubes: 20 μL, 40 μL and 60 μL of sample for 980 μL, 960 μL and 940 μL of methanol. Subsequently, an aliquot of 0.1 mL of each dilution of the extract was transferred to falcon tubes of 10 mL with 3.9 mL of the DPPH radical, and homogenized in a tube agitator. The samples were read at 515 nm in a UV-visible absorption spectrophotometer (Analytik Jena, Spekol 1300, Germany) after the reduction of absorbance until stabilization. Methyl alcohol was used as white point for calibration. The results were expressed as EC50 values in g of dry sample/g of DPPH.

##### Antioxidant Capacity by ABTS Free Radical Capture

Rufino et al. [24] described the technique to determine the antioxidant capacity by capturing ABTS*+. Three different dilutions of green, brown, and dark propolis extracts were prepared in triplicate: 20 μL, 40 μL and 60 μL of sample for 980 μL, 960 μL and 940 μL of ethyl alcohol. In falcon tubes, an aliquot of 30 μL of each extract dilution with 3.0 mL of the ABTS·+ radical was transferred and homogenized in a tube agitator. After incubation for 6 min, the absorbance reading was performed at 734 nm, using ethyl alcohol to calibrate the equipment.

##### Antioxidant Capacity by Iron Reduction Reaction (FRAP)

The FRAP method was performed as described by Rufino et al. [24]. Three different dilutions were prepared in falcon tubes of 10 mL, in triplicate, of green, brown, and dark propolis extracts: 20 μL, 40 μL and 60 μL of sample for 980 μL, 960 μL and 940 μL of distilled water. An aliquot of 90 μL of each dilution of the extract was transferred to falcon tubes, and 270 μL of distilled water was added. Subsequently, 2.7 mL of the FRAP reagent was added, and the mixture was homogenized in a tube agitator and kept in a water bath at 37 °C for 30 min. The absorbance reading was performed at 595 nm in a spectrophotometer, and the FRAP reagent was used as white point to calibrate the equipment.

#### 3.2.4. Chemical Profile Using Paper Spray Mass Spectrometry (PS-MS)

The chemical profile of green, brown, and dark propolis aqueous extracts was analyzed using an LCQ mass spectrometer (Thermo Scientific, San Jose, CA, USA) equipped with a paper spray ionization source. For the analysis, 2 μL of the samples and 40 μL of methanol were applied on chromatographic paper, and cut in triangular format (equilateral 1.5 cm) coupled to the equipment. The instrumental conditions of analysis were the following: PS-MS source voltage equal to −3.5 kV (negative ionization mode) and +4.5 kV (positive ionization mode), capillary voltage of 40 V, tube lens voltage of 120 V, transfer tube temperature of 275 °C, and mass range of 100 to 1000 for the positive and negative ionization modes. A comparison was made between the mass/charge ratios (*m*/*z*) obtained in the study with those found in the literature through fragmentation using sequential mass spectrometry, in order to identify the compounds under analysis. The collision energy used to fragment the compounds ranged from 15 to 40 V [78,79,80].

## 4. Conclusions

The green, brown and dark propolis extracts showed high antioxidant capacity and the presence of various phenolic compounds. In the studies analyzed, the green propolis extract demonstrated the highest content of total phenolic compounds and antioxidant capacity, followed by the brown and dark propolis.

The chemical profile of these extracts using paper spray ionization with mass spectrometry (PS-MS) allowed the possible identification of 116 compounds, including artepillin C, one of propolis’s main biologically active phenolic components.

The chemical profiles found in this study were similar to those reported in the literature for the three types of propolis. However, dark propolis is not extensively studied, demonstrating the relevance of this research when analyzing its chemical profile and identifying compounds found only in this type of propolis.

In addition, paper spray mass spectrometry to analyze the chemical profile of green, brown and dark propolis extracts, together with the analysis of antioxidant capacity and total phenolic compounds of these extracts, proved efficient in the evaluation of their chemical profiles, demonstrating the bioactive potential of the three extracts studied for application in food and pharmaceutical products.

The study of the chemical composition of propolis is essential to understand its biological capacity, establish correlations with its health benefits and promote the development of quality products based on science. This result contributes to propolis’s proper and safe use as a natural therapeutic resource.

## Figures and Tables

**Table 1:** Total Phenolic Compounds (TPC) and antioxidant capacity of propolis.

Samples	Analysis			
	TPC (mg GAE/100 g)	ABTS (Trolox μM/g)	FRAP (μM Ferrous Sulfate/g)	DPPH (EC50 Expressed in g of Sample/g of DPPH)
Green Propolis	2741.71 ± 49.53	293.90 ± 11.18	422.83 ± 21.42	491.68 ± 44.55
Brown Propolis	1191.55 ± 36.79	109.29 ± 10.37	179.54 ± 14.71	1054.38 ± 73.66
Dark Propolis	901.79 ± 27.80	162.57 ± 20.77	161.29 ± 2.59	1090.72 ± 55.28

EC50 = Amount of sample required to reduce the initial concentration of the DPPH radical by 50%. TPC = Total phenolic compounds. GAE = Gallic acid equivalent. Mean values ± standard deviation (*n* = 3).

## Data Availability

All data are contained within the article.

## Supplementary Materials

The following supporting information can be downloaded at: https://www.mdpi.com/article/10.3390/plants12183204/s1, Figure S1. PS(+)MS of the aqueous extract of the dark propolis; Figure S2. PS(+)MS of the aqueous extract of the green propolis; Figure S3. PS(+)MS of the aqueous extract of the brown propolis; Figure S4. PS(-)MS of the aqueous extract of the dark propolis; Figure S5. PS(-)MS of the aqueous extract of the green propolis; Figure S6. PS(-)MS of the aqueous extract of the brown propolis; Figure S7. Product ion mass spectrum (MS/MS) of the ion of *m/z* 301 (ascribed as protonated Artepillin C); Figure S8. Product ion mass spectrum (MS/MS) of the ion of *m/z* 317 (ascribed as protonated 7-hydroxy dehydroabietic acid); Figure S9. Product ion mass spectrum (MS/MS) of the ion of *m/z* 340 (ascribed as protonated Lobelanidin); Figure S10. Product ion mass spectrum (MS/MS) of the ion of *m/z* 163 (ascribed as deprotonated Coumaric p-acid); Figure S11. Product ion mass spectrum (MS/MS) of the ion of *m/z* 179 (ascribed as deprotonated Caffeic acid); Figure S12. Product ion mass spectrum (MS/MS) of the ion of *m/z* 231 (ascribed as deprotonated Drupanin); Figure S13. Product ion mass spectrum (MS/MS) of the ion of *m/z* 253 (ascribed as deprotonated Chrysin); Figure S14. Product ion mass spectrum (MS/MS) of the ion of *m/z* 255 (ascribed as deprotonated Pinocembrin); Figure S15. Product ion mass spectrum (MS/MS) of the ion of *m/z* 267 (ascribed as deprotonated Apigenin); Figure S16. Product ion mass spectrum (MS/MS) of the ion of *m/z* 269 (ascribed as deprotonated Caffeic Acid Benzyl Ester); Figure S17. Product ion mass spectrum (MS/MS) of the ion of *m/z* 271 (ascribed as deprotonated Naringenin); Figure S18. Product ion mass spectrum (MS/MS) of the ion of *m/z* 283 (ascribed as deprotonated Galangin-5-methyl-ether); Figure S19. Product ion mass spectrum (MS/MS) of the ion of *m/z* 289 (ascribed as deprotonated Epicatechin); Figure S20. Product ion mass spectrum (MS/MS) of the ion of *m/z* 295 (ascribed as deprotonated Kaempferide); Figure S21. Product ion mass spectrum (MS/MS) of the ion of *m/z* 301 (ascribed as deprotonated Quercetin); Figure S22. Product ion mass spectrum (MS/MS) of the ion of *m/z* 303 (ascribed as deprotonated Taxifolin); Figure S23. Product ion mass spectrum (MS/MS) of the ion of *m/z* 311 (ascribed as deprotonated Pinobanksin-3-acetate); Figure S24. Product ion mass spectrum (MS/MS) of the ion of *m/z* 313 (ascribed as deprotonated Quercetin 3-methyl ether); Figure S25. Product ion mass spectrum (MS/MS) of the ion of *m/z* 319 (ascribed as deprotonated Cupressic acid); Figure S26. Product ion mass spectrum (MS/MS) of the ion of *m/z* 333 (ascribed as deprotonated Agathic acid); Figure S27. Product ion mass spectrum (MS/MS) of the ion of *m/z* 353 (ascribed as deprotonated Chlorogenic acid); Figure S28. Product ion mass spectrum (MS/MS) of the ion of *m/z* 357 (ascribed as deprotonated Matairesinol); Figure S29. Product ion mass spectrum (MS/MS) of the ion of *m/z* 515 (ascribed as deprotonated Dicaffeoylquinic acid).
