# Supplementary material for: Evaluation of the Chemical Profile and Antioxidant Capacity of Green, Brown, and Dark Propolis
Source: Plants (Basel). 2023 Sep 8;12(18):3204. doi: 10.3390/plants12183204 (PMC10537587; doi:10.3390/plants12183204)

Supplementary file

Figure S1. PS(+)MS of the aqueous extract of the dark propolis.

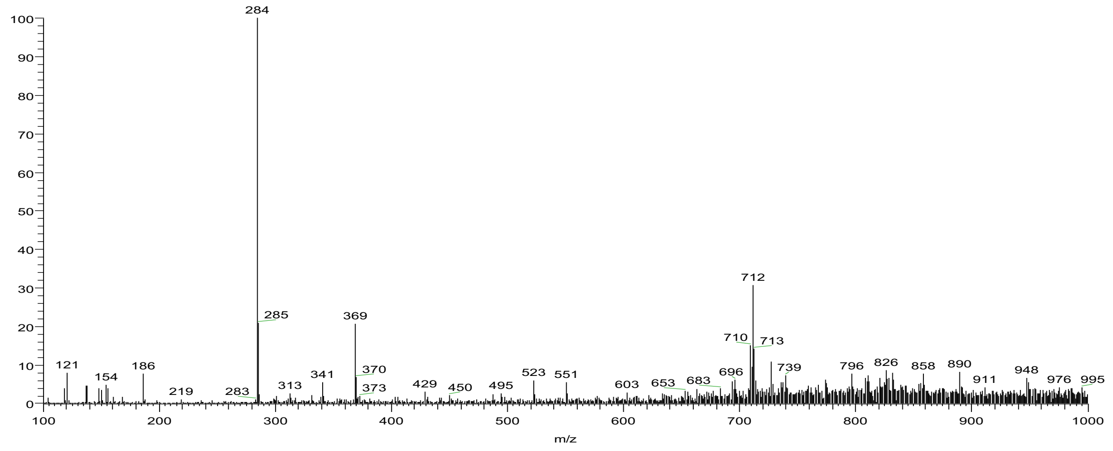

Figure S2. PS(+)MS of the aqueous extract of the green propolis.

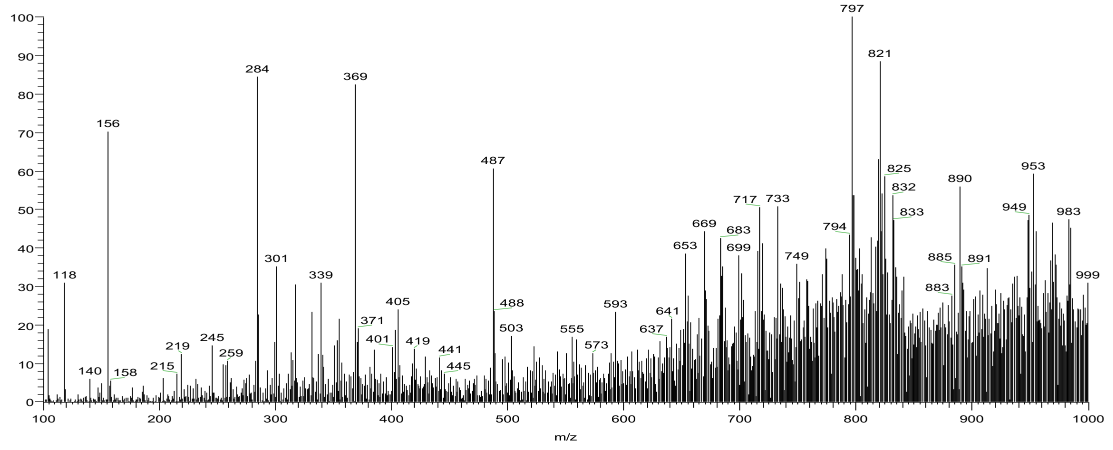

Figure S3. PS(+)MS of the aqueous extract of the brown propolis.

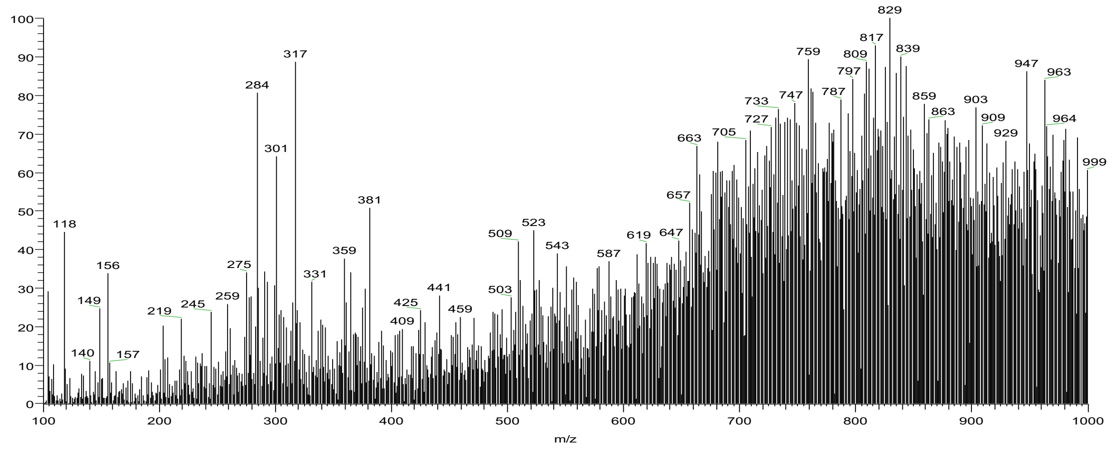

Figure S4. PS(-)MS of the aqueous extract of the dark propolis.

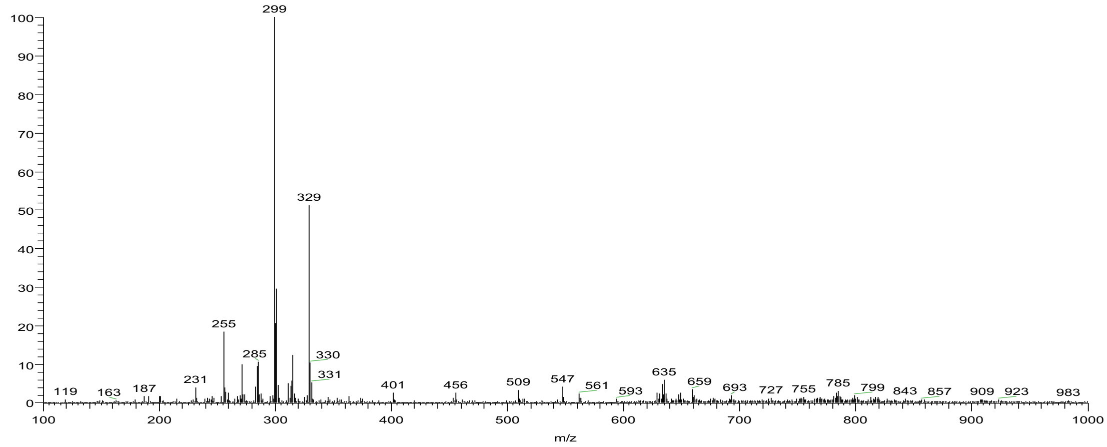

Figure S5. PS(-)MS of the aqueous extract of the green propolis.

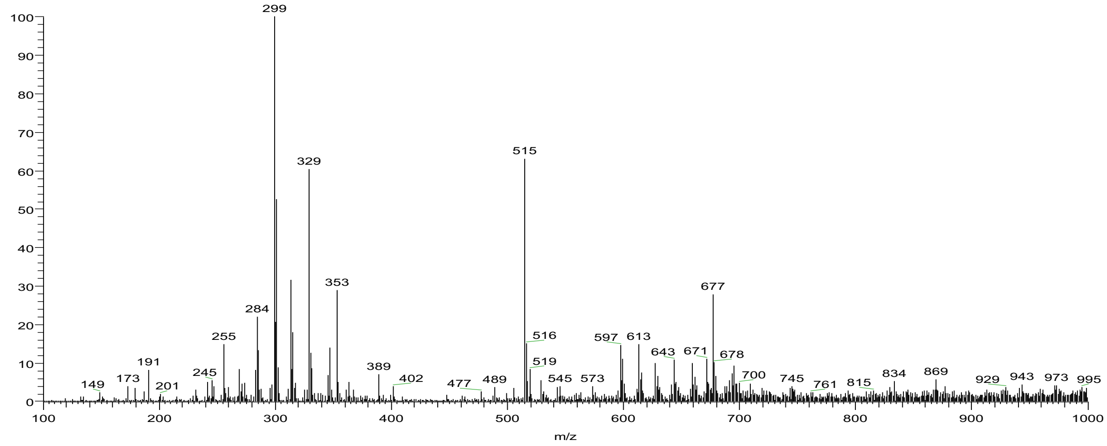

Figure S6. PS(-)MS of the aqueous extract of the brown propolis.

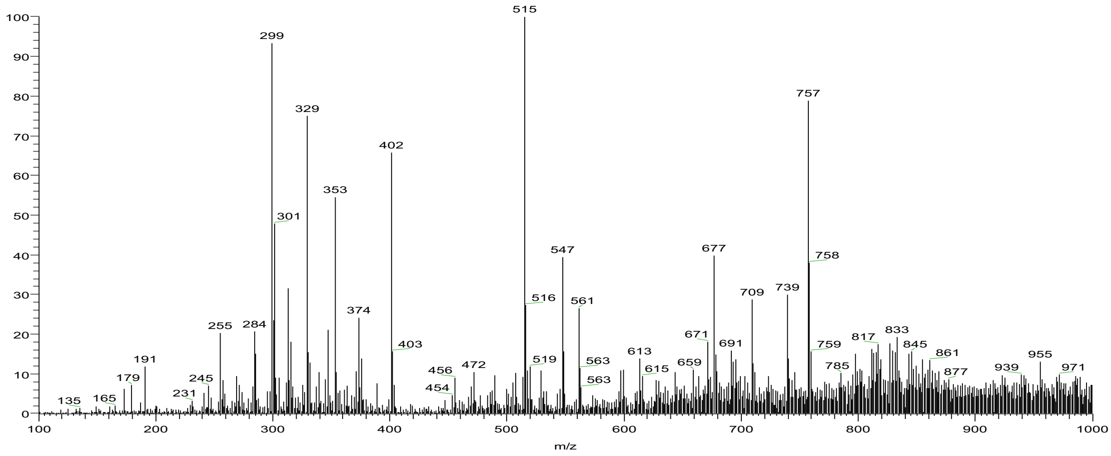

Figure S7. Product ion mass spectrum (MS/MS) of the ion of *m/z* 301 (ascribed as protonated Artepillin C).

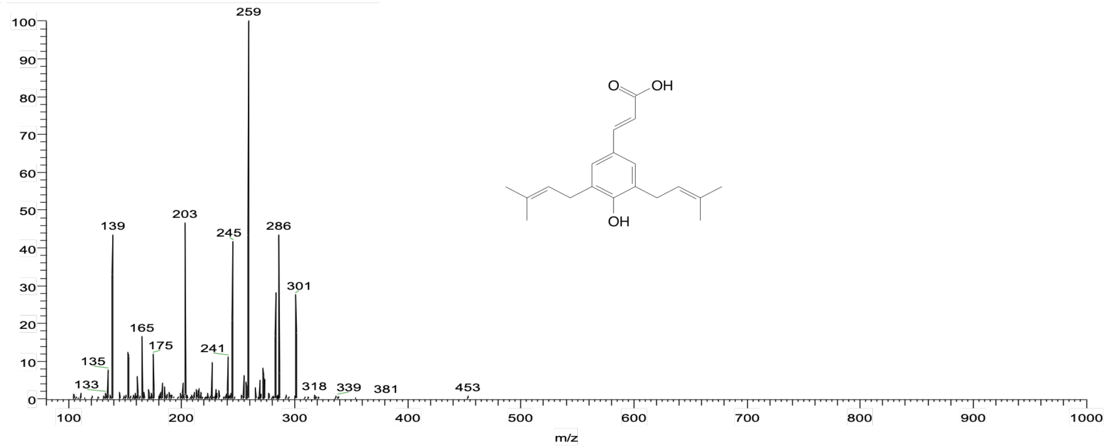

Figure S8. Product ion mass spectrum (MS/MS) of the ion of *m/z* 317 (ascribed as protonated 7-hydroxy dehydroabietic acid).

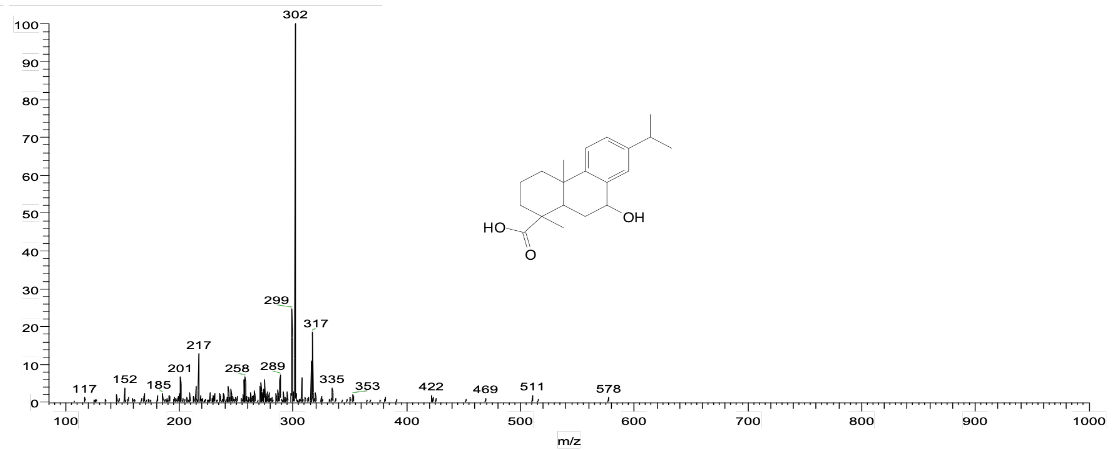

Figure S9. Product ion mass spectrum (MS/MS) of the ion of *m/z* 340 (ascribed as protonated Lobelanidin).

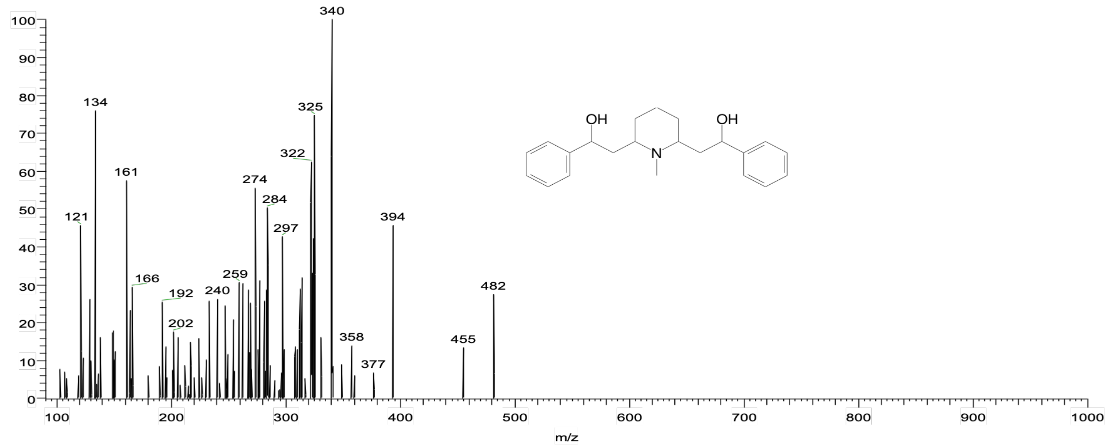

Figure S10. Product ion mass spectrum (MS/MS) of the ion of *m/z* 163 (ascribed as deprotonated Coumaric *p*-acid).

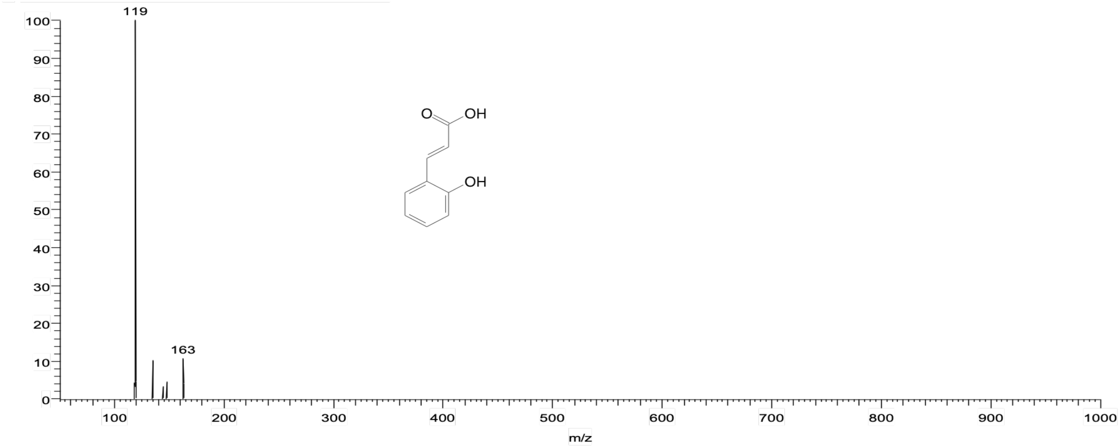

Figure S11. Product ion mass spectrum (MS/MS) of the ion of *m/z* 179 (ascribed as deprotonated Caffeic acid).

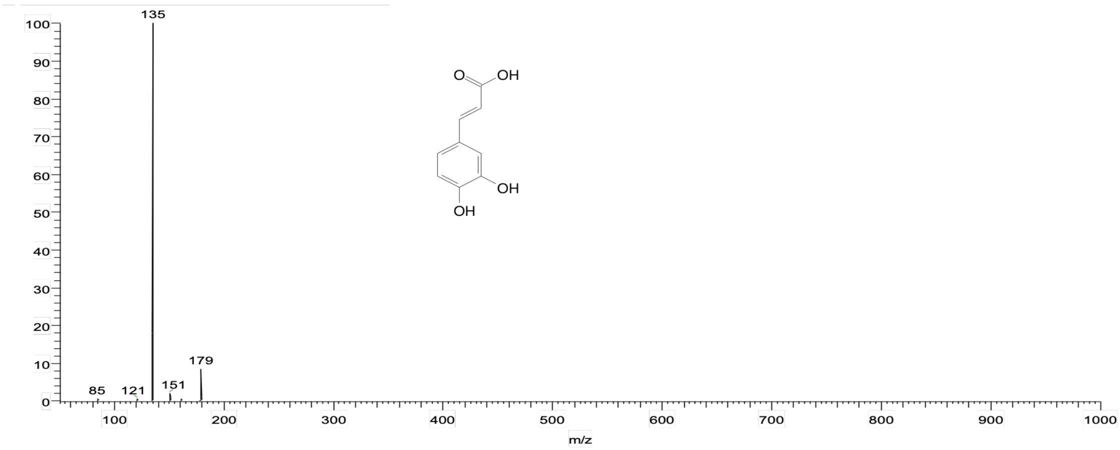

Figure S12. Product ion mass spectrum (MS/MS) of the ion of *m/z* 231 (ascribed as deprotonated Drupanin).

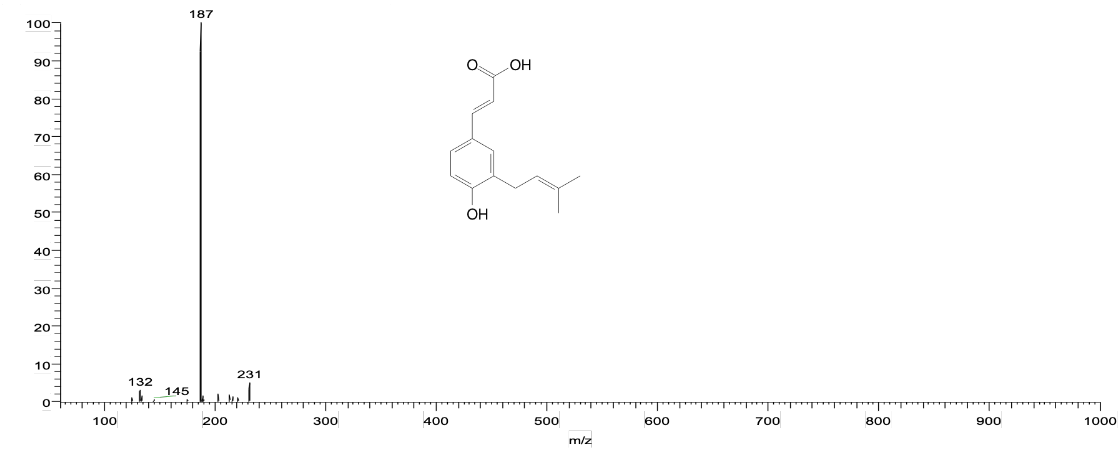

Figure S13. Product ion mass spectrum (MS/MS) of the ion of *m/z* 253 (ascribed as deprotonated Chrysin).

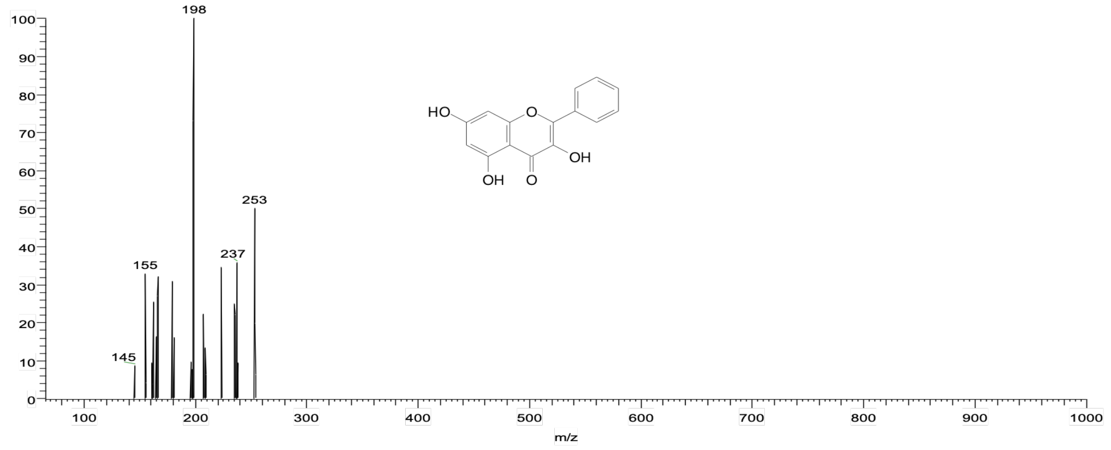

Figure S14. Product ion mass spectrum (MS/MS) of the ion of *m/z* 255 (ascribed as deprotonated Pinocembrin).

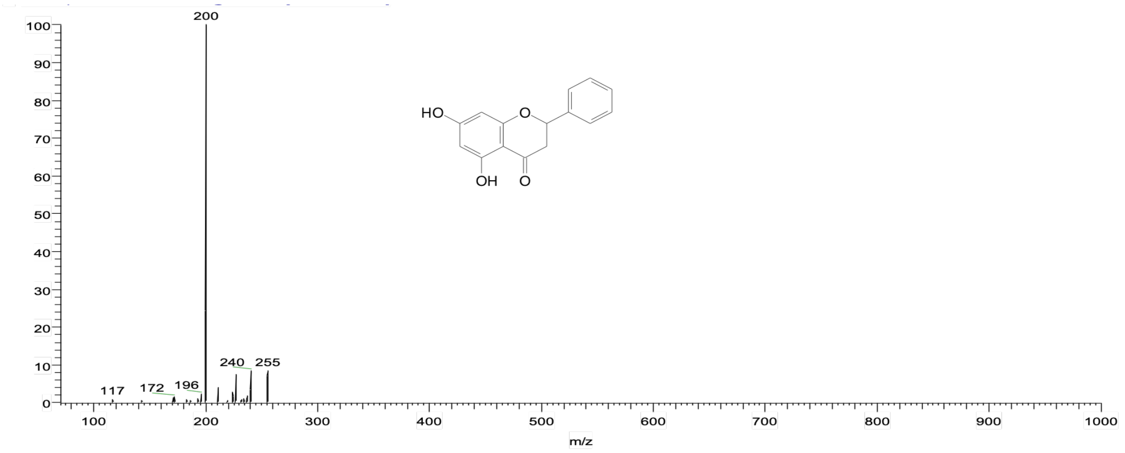

Figure S15. Product ion mass spectrum (MS/MS) of the ion of *m/z* 267 (ascribed as deprotonated Apigenin).

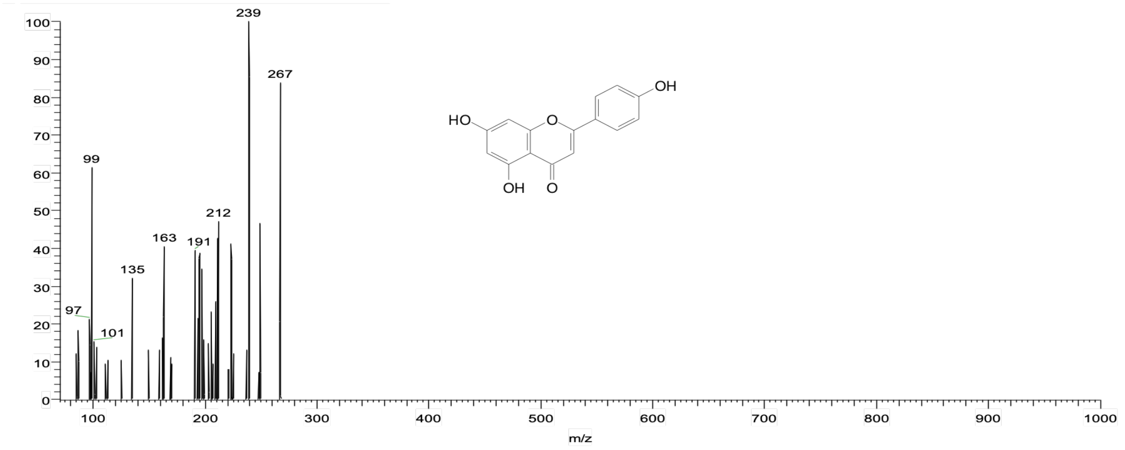

Figure S16. Product ion mass spectrum (MS/MS) of the ion of *m/z* 269 (ascribed as deprotonated Caffeic Acid Benzyl Ester).

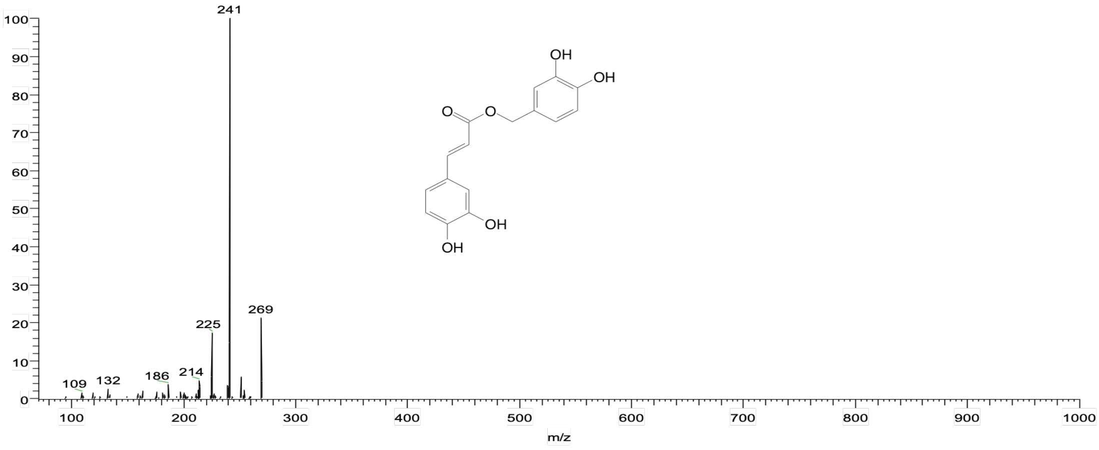

Figure S17. Product ion mass spectrum (MS/MS) of the ion of *m/z* 271 (ascribed as deprotonated Naringenin).

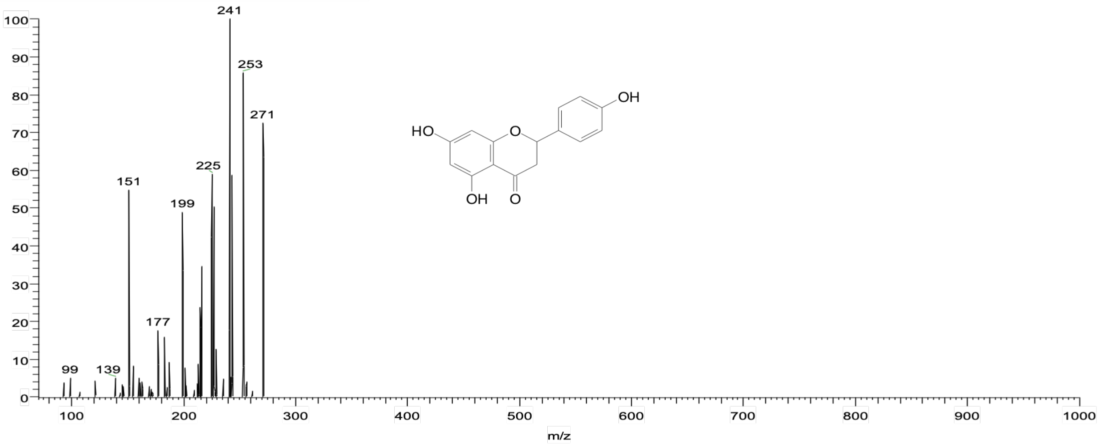

Figure S18. Product ion mass spectrum (MS/MS) of the ion of *m/z* 283 (ascribed as deprotonated Galangin-5-methyl-ether).

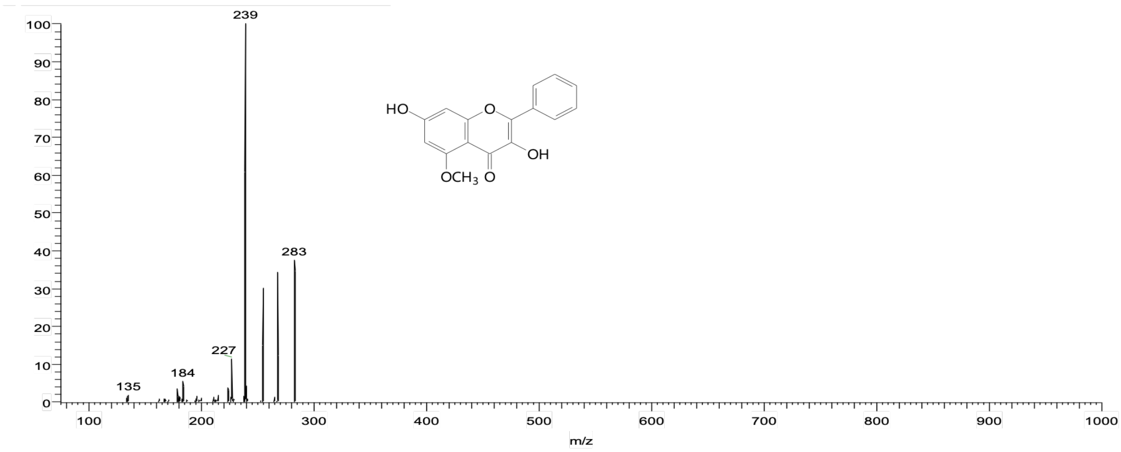

Figure S19. Product ion mass spectrum (MS/MS) of the ion of *m/z* 289 (ascribed as deprotonated Epicatechin).

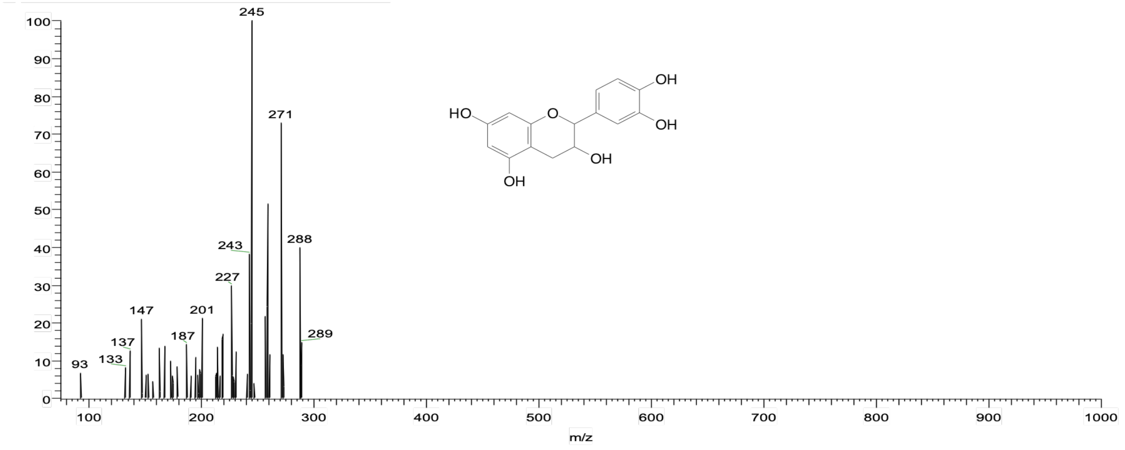

Figure S20. Product ion mass spectrum (MS/MS) of the ion of *m/z* 295 (ascribed as deprotonated Kaempferide).

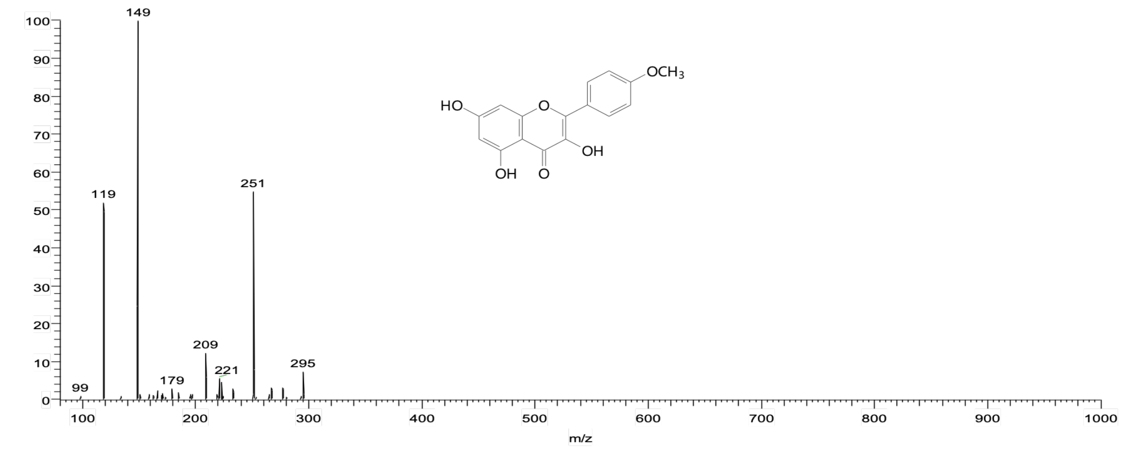

Figure S21. Product ion mass spectrum (MS/MS) of the ion of *m/z* 301 (ascribed as deprotonated Quercetin).

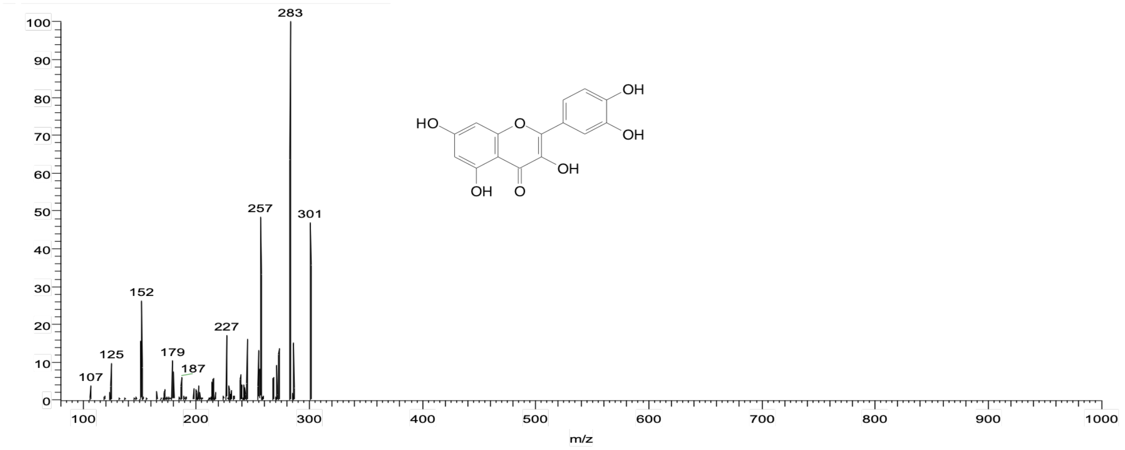

Figure S22. Product ion mass spectrum (MS/MS) of the ion of *m/z* 303 (ascribed as deprotonated Taxifolin).

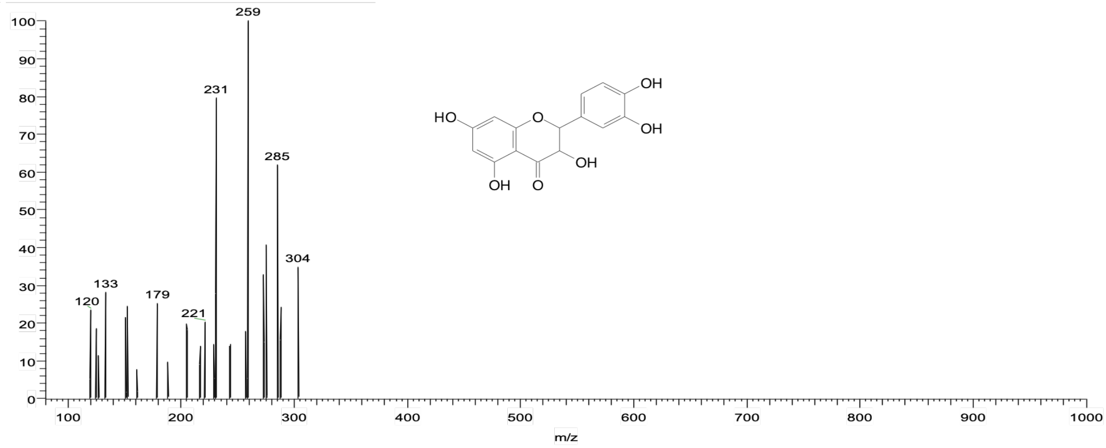

Figure S23. Product ion mass spectrum (MS/MS) of the ion of *m/z* 311 (ascribed as deprotonated Pinobanksin-3-acetate).

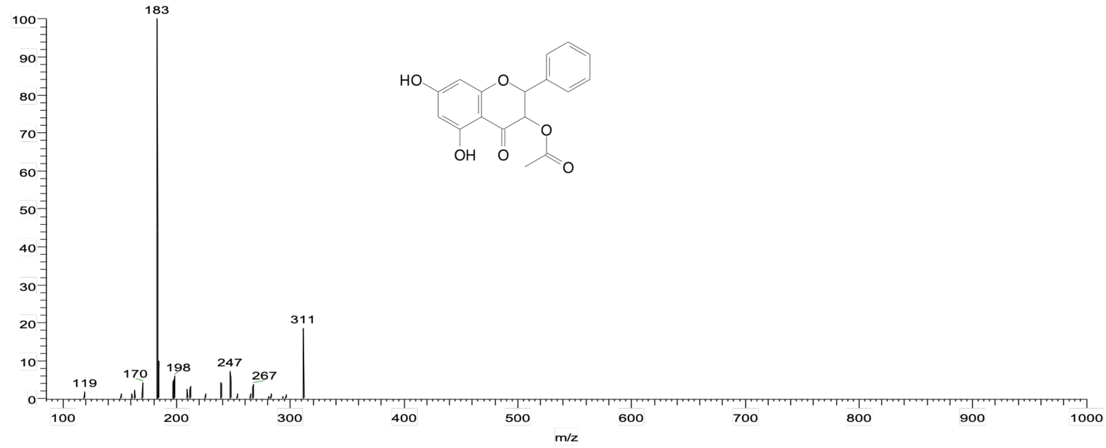

Figure S24. Product ion mass spectrum (MS/MS) of the ion of *m/z* 313 (ascribed as deprotonated Quercetin 3-methyl ether).

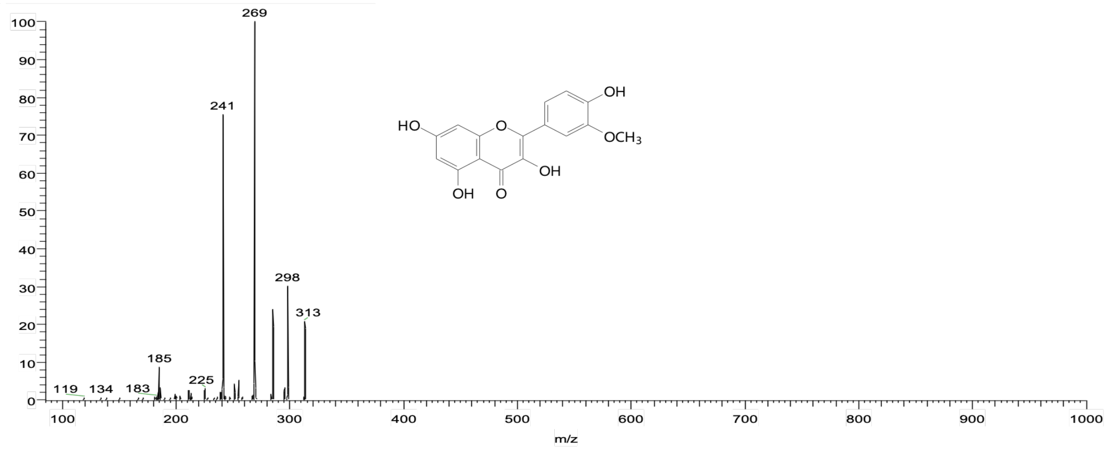

Figure S25. Product ion mass spectrum (MS/MS) of the ion of *m/z* 319 (ascribed as deprotonated Cupressic acid).

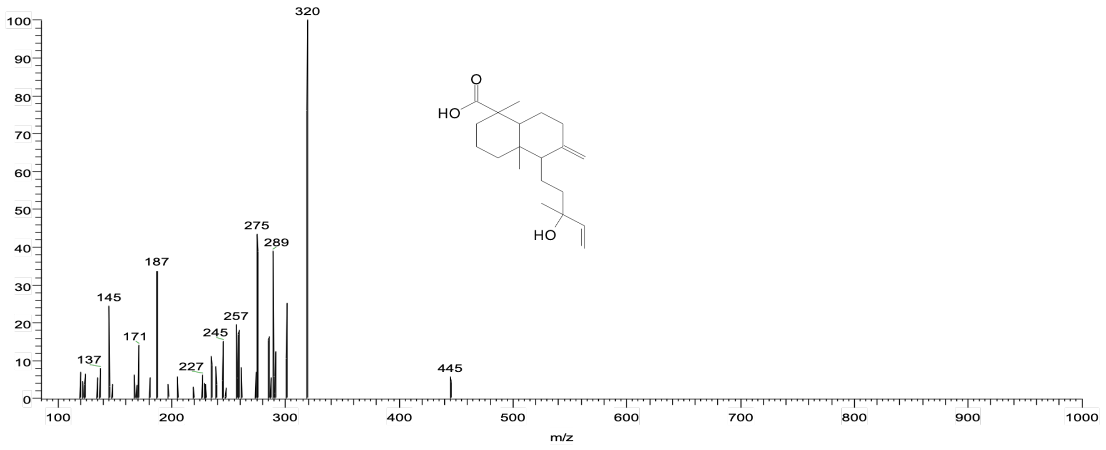

Figure S26. Product ion mass spectrum (MS/MS) of the ion of *m/z* 333 (ascribed as deprotonated Agathic acid).

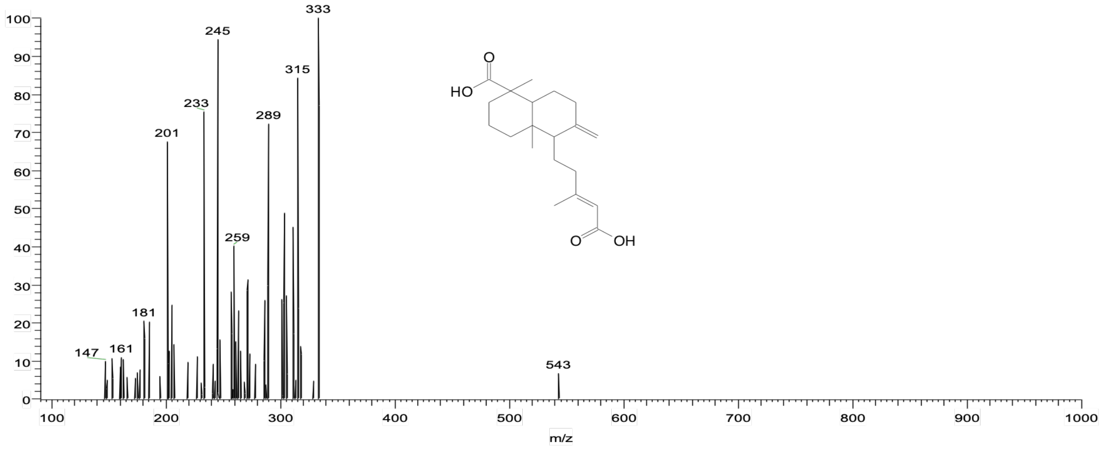

Figure S27. Product ion mass spectrum (MS/MS) of the ion of *m/z* 353 (ascribed as deprotonated Chlorogenic acid).

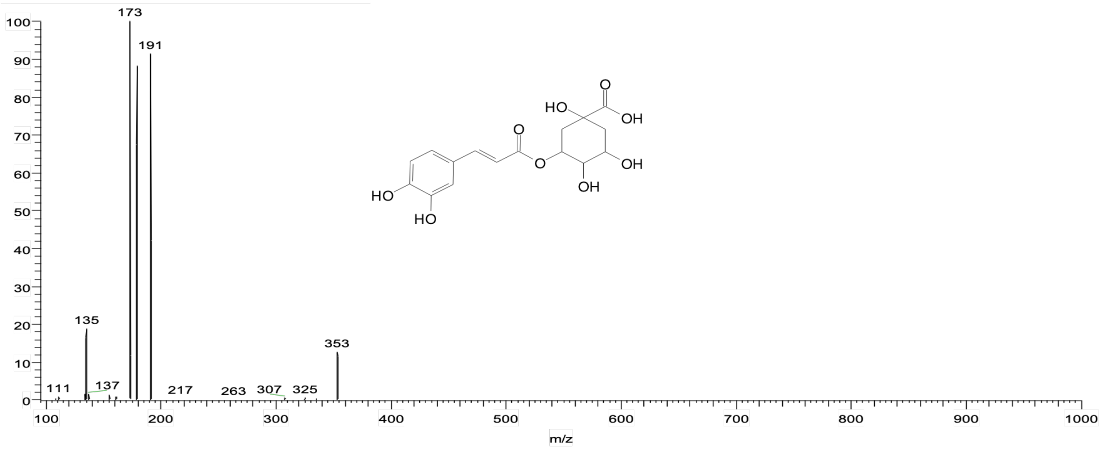

Figure S28. Product ion mass spectrum (MS/MS) of the ion of *m/z* 357 (ascribed as deprotonated Matairesinol).

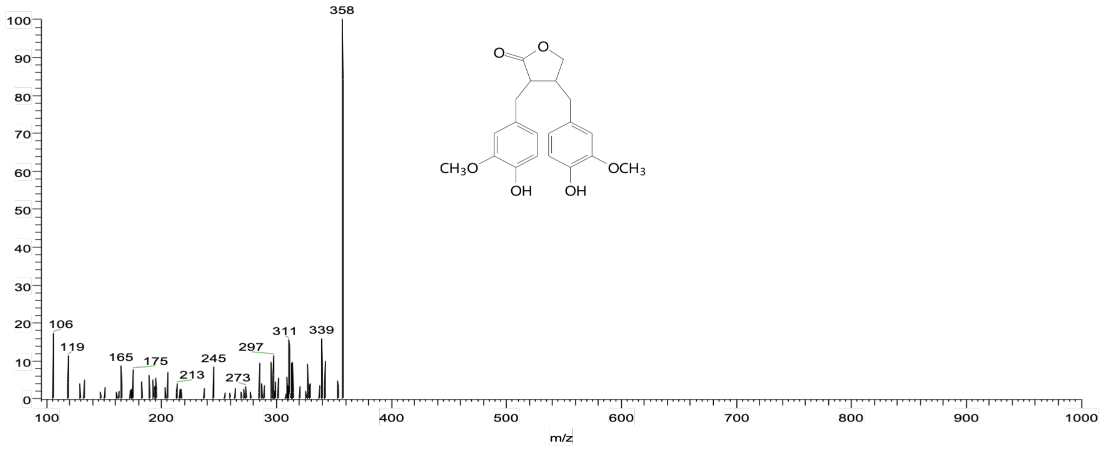

Figure S29. Product ion mass spectrum (MS/MS) of the ion of *m/z* 515 (ascribed as deprotonated Dicaffeoylquinic acid).

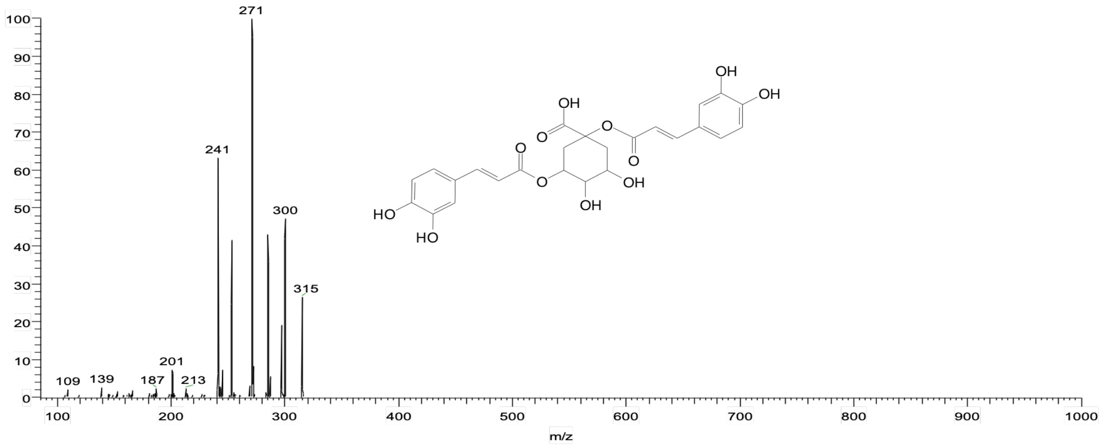

Supplement: Supplementary file 1 [file plants-12-03204-s001.zip › plants-2509746-supplementary.pdf]
